# Effectiveness of the physical activity intervention program in the PREDIMED-Plus study: a randomized controlled trial

**DOI:** 10.1186/s12966-018-0741-x

**Published:** 2018-11-13

**Authors:** Helmut Schröder, Gabriela Cárdenas-Fuentes, Miguel Angel Martínez-González, Dolores Corella, Jesús Vioque, Dora Romaguera, J. Alfredo Martínez, Francisco J. Tinahones, José López Miranda, Ramon Estruch, Aurora Bueno-Cavanillas, Fernando Arós, Ascensión Marcos, Josep A. Tur, Julia Warnberg, Lluis Serra-Majem, Vicente Martín, Clotilde Vázquez, José Lapetra, Xavier Pintó, Josep Vidal, Lidia Daimiel, José Juan Gaforio, Pilar Matía-Martín, Emilio Ros, Olga Castañer, Camille Lassale, Miguel Ruiz-Canela, Eva M. Asensio, Josep Basora, Laura Torres-Collado, Antonio Garcia-Rios, Itziar Abete, Estefania Toledo, Pilar Buil-Cosiales, Mònica Bullo, Albert Goday, Montserrat Fitó, Jordi Salas-Salvadó, On behalf of the PREDIMED-Plus investigators

**Affiliations:** 1 Cardiovascular Risk and Nutrition Research Group (CARIN), Hospital del Mar, Medical Research Institute (IMIM), Barcelona, Spain; 2 CIBER Epidemiologia y Salud Pública (CIBERESP), Instituto de Salud Carlos III, Madrid, Spain; 3 Department of Experimental and Health Sciences, Universidad Pompeu Fabra, Barcelona, Spain; 4 Department of Preventive Medicine and Public Health, University of Navarra-Navarra Institute for Health Research, Pamplona, Spain; 5 CIBER de Fisiopatología de la Obesidad y la Nutrición (CIBEROBN), Instituto de Salud Carlos III, Madrid, Spain; 6 Department of Preventive Medicine, University of Valencia, Valencia, Spain; 7 Nutritional Epidemiology Unit, Miguel Hernández University, ISABIAL-FISABIO, Alicante, Spain; 8 Instituto de Investigación Sanitaria Illes Balears (IdISPa), Hospital Universitario Son Espases, Mallorca, Spain; 9 Department of Nutrition, Food Sciences, and Physiology, Center for Nutrition Research, University of Navarra, Pamplona, Spain; 10 Madrid Institute for Advanced Studies (IMDEA) Food Institute, Madrid, Spain; 11 Department of Endocrinology and Nutrition, Virgen de la Victoria Hospital, Malaga University, Malaga, Spain; 12 Lipids and Atherosclerosis Unit, Department of Internal Medicine, Maimonides Biomedical Research Institute of Cordoba (IMIBIC), Reina Sofia University Hospital, University of Cordoba, Córdoba, Spain; 13 Department of Internal Medicine, Hospital Clínic, IDIBAPS August Pi i Sunyer Biomedical Research Institute, University of Barcelona, Barcelona, Spain; 14 Departament of Preventive Medicine and Public Health, University of Granada, Granada, Spain; 15 OSI ARABA. University Hospital Araba, Department of Cardiology, University of the Basque Country UPV/EHU Vitoria-Gasteiz, Vitoria-Gasteiz, Spain; 16 Immunonutrition Research Group, Department Metabolism and Nutrition, Institute of Food Science, Technology and Nutrition (ICTAN), Spanish National Research Council (CSIC), Madrid, Spain; 17 Research Group on Community Nutrition and Oxidative Stress, University of the Balearic Islands, Palma de Mallorca, Spain; 18 Department of Nursing, School of Health Sciences, University of Málaga, Málaga, Spain; 19 Research Institute of Biomedical and Health Sciences, University of Las Palmas de Gran Canaria, Las Palmas de Gran Canaria, Spain; 20 Institute of Biomedicine (IBIOMED), University of León, León, Spain; 21 Department of Endocrinology and Nutrition, University Hospital Fundación Jiménez Díaz, Madrid, Spain; 22 Department of Family Medicine, Research Unit, Distrito Sanitario Atención Primaria Sevilla, Sevilla, Spain; 23 Lipid Unit, Department of Internal Medicine, Bellvitge Biomedical Research Institute (IDIBELL)-Hospital Universitari de Bellvitge, L’Hospitalet de Llobregat, Barcelona Spain; 24 Department of Endocrinology and Nutrition, Hospital Clínic, Barcelona, Spain; 25 CIBER de Diabetes y Enfermedades Metabólicas Asociadas (CIBERDEM), Instituto de Salud Carlos III, Madrid, Spain; 26 Nutritional Genomics and Epigenomics group, Madrid Institute for Advanced Studies (IMDEA) Food Institute, CEI UAM + CSIC, Madrid, Spain; 27 Center for Advanced Studies in Olive Grove and Olive Oils, University of Jaen, Jaen, Spain; 28 Department of Endocrinology and Nutrition, Hospital Clínico San Carlos, Instituto de Investigación Sanitaria San Carlos (IDISSC), Madrid, Spain; 29 Department of Lipids, Hospital Clínic, Institut d’Investigacions Biomediques August Pi i Sunyer (IDIBAPS), University of Barcelona, Barcelona, Spain; 30 Research Department of Epidemiology & Public Health, University College London, London, UK; 31 Servicio Navarro de Salud, Primary Health Care, Pamplona, Spain; 32 Department of Biochemistry and Biotechnology, Human Nutrition Unit, Hospital Universitari Sant Joan de Reus, Reus, Spain; 33 Department of Endocrinology and Nutrition, Hospital del Mar, Barcelona, Spain; 34 Human Nutrition Unit, University Hospital of Sant Joan de Reus, Reus, Spain; 35 Department of Biochemistry and Biotechnology, Pere Virgili Institute for Health Research, Rovira i Virgili University, Reus, Spain

**Keywords:** Randomized control trial, Physical activity, Older adults, Intervention program, Body mass index, Waist circumference

## Abstract

**Background:**

The development and implementation of effective physical activity (PA) intervention programs is challenging, particularly in older adults. After the first year of the intervention program used in the ongoing PREvención con DIeta MEDiterránea (PREDIMED)-Plus trial, we assessed the initial effectiveness of the PA component.

**Methods:**

PREDIMED-Plus is an ongoing randomized clinical trial including 6874 participants randomized to an intensive weight-loss lifestyle intervention based on an energy-restricted Mediterranean diet (MedDiet), physical activity promotion and behavioral support and to a control group using MedDiet recommendations but without calorie restriction or PA advice. Body mass index (BMI) and waist circumference (WC) are measured by standard clinical protocols. Duration and intensity of PA is self-reported using the validated REGICOR Short Physical Activity Questionnaire. The primary endpoint of the PREDIMED-Plus trial is a combined cardiovascular outcome: myocardial infarction (acute coronary syndromes with positive troponin test), stroke, or cardiovascular mortality. The present study involved secondary analysis of PA data (*n* = 6059; mean age 65 ± 4.9 years) with one-year changes in total, light, and moderate-to-vigorous PA within and between intervention groups as the outcome. Generalized estimating equation models were fitted to evaluate time trends of PA, BMI, and WC within groups and differences between intervention and control groups.

**Results:**

After 12 months, average daily MVPA increased by 27.2 (95%CI 5.7;48.7) METs-min/day and 123.1 (95%CI 109.7–136.6) METs-min/day in the control and intervention groups, respectively. Total-PA, light-PA, and MVPA increased significantly (*p* < 0.01) in both groups. A significant (*p* < 0.001) time*intervention group interaction was found for Total-PA and MVPA, meaning the PA trajectory over time differed between the intervention and control groups. Age, sex, education level, and BMI did not moderate the effectiveness of the PA intervention. BMI and WC decreased significantly with increasing MVPA, compared with participants who reported no changes in MVPA.

**Conclusion:**

After one year of follow-up, the PREDIMED-Plus PA intervention has been effective in increasing daily PA in older adults.

**Trial registration:**

Retrospectively registered at the International Standard Randomized Controlled Trial (http://www.isrctn.com/ISRCTN89898870), registration date: 24 July 2014.

## Background

Regular physical activity (PA) is associated with numerous health benefits [1, 2]. Adherence to PA recommendations is associated with a significant reduction in cardiovascular disease and all-cause mortality [3].

Engagement in PA, especially at moderate and high intensities, decreases with ageing [4, 5], whereas multimorbidity increases [6]. Additionally, sedentary behaviors are highly prevalent in older adults [7]. The increase in sedentary behaviors and the concurrent decrease in PA in older adults are positively associated with weight gain and an increased incidence of obesity and obesity-related comorbidities [8, 9]. Therefore, it is paramount to implement PA intervention programs to improve physical and mental health in this growing segment of contemporary society.

A recently published meta-analysis [10] of the effectiveness of PA interventions in older adults showed a moderate effect size of a difference of 73 min per week in favor of the intervention group compared with the control group. Whether this effect size is sufficient to promote a clinically significant reduction in weight gain is questionable [11]. In contrast, short-term interventions with multiple structured and controlled sessions of moderate to vigorous exercise per week have a higher effect size regarding PA increase in the intervention group, and support weight loss and a reduction in waist circumference (WC) in older adults [12]. However, maintaining such an intervention strategy is hardly feasible in the long run.

The present study assessed the effectiveness at one year of a PA intervention program included as one of the main aspects of the multilevel intervention in the ongoing PREDIMED-Plus primary prevention randomized trial, designed for older individuals at high risk for cardiovascular disease (CVD). We gave special attention to potential effect moderators, in particular sex, age, education, and obesity. Finally, we evaluated the one-year change in PA and changes in body mass index (BMI) and WC.

## Methods

### Study design

PREDIMED-Plus is a six-year, multicenter, parallel-group, randomized trial. Details on the protocol can be found at http://predimedplus.com/ [13]. The ongoing PREDIMED-Plus trial was registered at the International Standard Randomized Controlled Trial (http://www.isrctn.com/ISRCTN89898870; registration date, 24 July 2014) [14]. From October 2013 to December 2016, 6874 participants were recruited from 23 Spanish centers. Participants were randomly assigned, in a 1:1 ratio, to one of two groups: an intensive weight-loss intervention group (based on a Mediterranean diet (MedDiet) with energy restrictions, individualized PA promotion, and behavioral support) or a control group, which included an unrestricted-energy MedDiet and traditional health care. The primary endpoint of the ongoing PREDIMED-Plus trial is a combined cardiovascular outcome: myocardial infarction (acute coronary syndromes with positive troponin test), stroke, or cardiovascular mortality. The present study involved a secondary analysis of the PA data; this was not pre-specified in the PREDIMED-Plus trial protocol. The primary outcome in this analysis was the change in total-PA, light-PA, and moderate-to-vigorous PA, assessed within and between intervention groups. The analysis was a partial intention-to-treat analysis (PITT) with treatment group membership as per random allocation, but including only trial participants with complete one-year follow-up data [15] related to the primary outcome of the present study (*n* = 6059).

### Participants

Eligible participants were men (aged 55–75 years) and women (aged 60–75 years) at high risk of CVD. The inclusion criteria were overweight or obesity (BMI ≥27 and < 40 kg/m2) and the presence of metabolic syndrome (i.e., fulfilling at least three of the metabolic dysfunction criteria defined by the International Diabetes Federation, American Heart Association, and National Heart, Lung, and Blood Institute [16]. Exclusion criteria included previous history of cardiovascular disease, any chronic medical condition (cancer, inflammatory bowel disease, cirrhosis, etc.), acute infectious processes, institutionalization, psychiatric disorders, any condition inhibiting PA, alcohol and drug abuse, use of specific medications (cytotoxic agents, immune-suppressors, etc.), important weight loss within a short time-period, and any allergy to MedDiet foods. Data were recorded in each of the 23 centers of the ongoing PREDIMED-Plus trial. Research Ethics Committees from all 23 recruitment centers approved the protocol for the present study, according to the ethical standards of the Declaration of Helsinki and all participants provided written informed consent. This study followed the CONSORT guidelines for reporting [17].

### Intervention

The ongoing PREDIMED-Plus trial delivers a dietary and PA intervention aimed to promote weight loss and reduce hard cardiovascular events (http://predimedplus.com/) [13]. Energy restriction and an increase in PA are essential to achieve weight loss. The PA intervention is a face-to-face tailored intervention program including goal setting (BCT taxonomy1.1) [18], action planning (BCT taxonomy 1.4), feedback (BCT taxonomy 2.2), informational materials, motivation, and self-monitoring (BCT taxonomy 2.3). During the first year of the ongoing trial (the time period analyzed in the present study), participants in the intervention group received PA recommendations by means of a tailored face-to-face educational program including 12 individual one-hour sessions, 12 telephone calls, and 3 one-hour group sessions focused on PA. The program was delivered by dietitians who received additional training in PA recommendations. The one-year retention rate was high (89.7%).

During the individual face-to-face sessions in each of the 23 centers participating in the trial, the dietitians explained to each participant the health benefits of being physically active (BCT taxonomy 5.1). Together, they set tailored PA goals and an action plan, taking into account individual preferences and possibilities.

During the first 6 months of intervention, participants were encouraged to gradually increase their activity level to at least 150 min/week of moderate-to-vigorous PA (MVPA), with the ultimate goal of walking at least 45 min per day, 6 days per week, and doing static exercises to improve strength, flexibility, and balance according to specific instructions. In each individual session with the dietitian, feedback (BCT taxonomy 2.2) was provided on progress toward personal goals associated with other activities that improve strength, resistance, balance, and flexibility. Participants could discuss difficulties in completing individual goals with the dietitians, who provided options tailored to each participant (BCT taxonomy 1.2). In addition, videos and brochures were provided and discussed, and a monthly motivational phone call from the dietitian reinforced PA goals.

Due to the specific characteristics of the study population, goal-setting for aerobic PA was mainly based on walking. Participants received a pedometer (Yamax SW200 Digi-Walker) and a PA diary as self-monitoring and motivational tools. In each individual visit with the dietitian, participants were encouraged to continue a progressive increase in their level of PA. Fidelity to intervention adherence was measured by periodically administered REGICOR Short Physical Questionnaire (RSPAQ) and pedometers. Additionally, all participants in both groups received free virgin olive oil (6 l every 6 months) and nuts (3 kg every 6 months) to increase adherence to the PREDIMED-Plus protocol. Advice on PA was not given to the control group.

### Measurements

At baseline, a general questionnaire [19] was used to record socio-demographic variables, smoking status, medical history, and use of medication. Education level was dichotomized as having more or less than a primary education.

MedDiet adherence was measured by a 14-item diet questionnaire, previously validated [20]. A 17-item questionnaire was used to assess adherence to the energy-restricted MedDiet and a 143-item food frequency questionnaire [21] to measure energy intake. These three questionnaires were completed at baseline and after 6 and 12 months of follow-up. In the ongoing trial, these data are being collected annually.

### Outcomes

The primary outcome of the present study, change in PA duration and intensity, was measured using the validated RSPAQ [22] at baseline and at 6 and 12 months. The main construct of this questionnaire covers all four dimensions of PA: type of activity, frequency, duration, and intensity. The questionnaire lists 6 types of activities: walking, brisk walking, walking on trails/hiking, gardening, climbing stairs, and sport activities. To complete the questionnaire, trained personnel asked participants the number of days per month and the average minutes per day they performed the activity.

The validation study of the RSPAQ [22] revealed a high reliability (intraclass correlation coefficient for total-PA = 0.82) and a reasonable validity (Spearman correlation coefficient for total-PA = 0.39). Additionally, the RSPAQ was sensitive in detecting changes in moderate and vigorous PA from baseline to the last visit analyzed (week 27). The Spearmen correlation coefficients between changes in PA derived by the RSPAQ and by accelerometers were 0.34 (*p* = 0.001) and 0.28 (*p* = 0.008) for moderate and vigorous PA, respectively.

Total energy expenditure in PA was estimated in Metabolic Equivalent of Tasks (METs)/min/day. An intensity code was assigned to each activity according to the Compendium of Physical Activities [23]. The METs assigned to each activity were then multiplied by the number of times per month and by the minutes per day the activity was performed. Finally, the values obtained were divided by 30 (days). PA was further classified according to intensity: light (< 4 METs), moderate (4–5.5 METs), and vigorous (≥6 METs).

Secondary outcomes of the present study were changes in body mass index (BMI) and WC. Anthropometric variables (height, weight, WC) were directly measured using a wall-mounted stadiometer, electronic scale, and anthropometric tape, respectively. The BMI was calculated by dividing weight (kg) by the height squared (m^2^). Obesity was defined as BMI ≥30 kg/m^2^.

### Sample size and randomization

Assuming a two-tailed alpha error of 0.05, a cumulative incidence in the control group after 6 years of at least 10%, an anticipated hazard ratio (HR) for the combined primary cardiovascular end-point of 0.70, and dropout rates of up to 20%, the required sample size was approximately 1600 participants per group. To be conservative, we aimed to recruit at least 6000 study participants (3000 in each group). Sample size calculation was performed for the primary outcome of the PREDIMED-Plus trial but not for secondary data analysis. Nonetheless, the secondary analysis is more than 99% powered for the main finding of the present study: daily average MVPA increased 123.1 (95%CI 109.7–136.6) METs-min/day between baseline and follow-up in the intervention group. Applying a potential drop-out rate of 20% and accepting an alpha risk of 0.05 and a beta risk of 0.2 in a two-sided test, 91 subjects were needed to recognize as statistically significant a difference greater than or equal to 0.05 units.

To assess willingness to participate in the study and to predict adherence to the intended intervention, participants attended a screening visit followed by a four-week run-in period before randomization. Study participants were randomized 1:1 into two groups; this procedure was blinded to all staff members and investigators. The random allocation was centralized and internet-based, generating blocks of 6 subjects stratified by sex, age (< 65, 65–70, > 70), and participating center. Spouses who wished to belong to the same group were randomized as a unit; this was the case for 806 participants (403 couples). Participants at each of the 23 centers received the information about their group allocation in the baseline visit.

### Statistical analysis

Baseline characteristics of the study population are presented as means ± standard deviations (SD) or median and interquartile range for quantitative variables, and as numbers and percentages for categorical variables. Student t, Mann–Whitney U, and Chi-square tests were used to determine differences in baseline characteristics between the intervention and control groups.

Generalized estimating equation (GEE) models were used to asses i) time trends of BMI, WC, total-PA, light-PA, and MVPA in each group and ii) differences between the intervention and control groups in these same variables, taking into account repeated measurements in each participant. GEE models were also fitted for the analysis of total PA, light PA, and MVPA, stratified by the following potential moderators: sex (male/female), age (< 65 years vs ≥65 years), educational level (primary vs more than primary education), and BMI (< 30 kg/m^2^ vs ≥30 kg/m^2^).

Finally, we conducted multiple linear regression analysis with cubic spline modeling to determine dose-response associations between changes in MVPA and changes in BMI and WC in the control and the intervention group, using the ‘gam’ package in R version 3.0.2. No change in MVPA was set as the reference value. Models were adjusted for age, sex, education, smoking, changes in MedDiet adherence, and the corresponding anthropometric baseline value.

Associations were considered statistically significant if *P* < 0.05. The SPSS for Windows version 21 (IBM Corp.: Armonk, NY, United States) and R-project, version 3.0.2 (R Foundation for Statistical Computing) were used for statistical analysis.

## Results

**Fig. 1:**
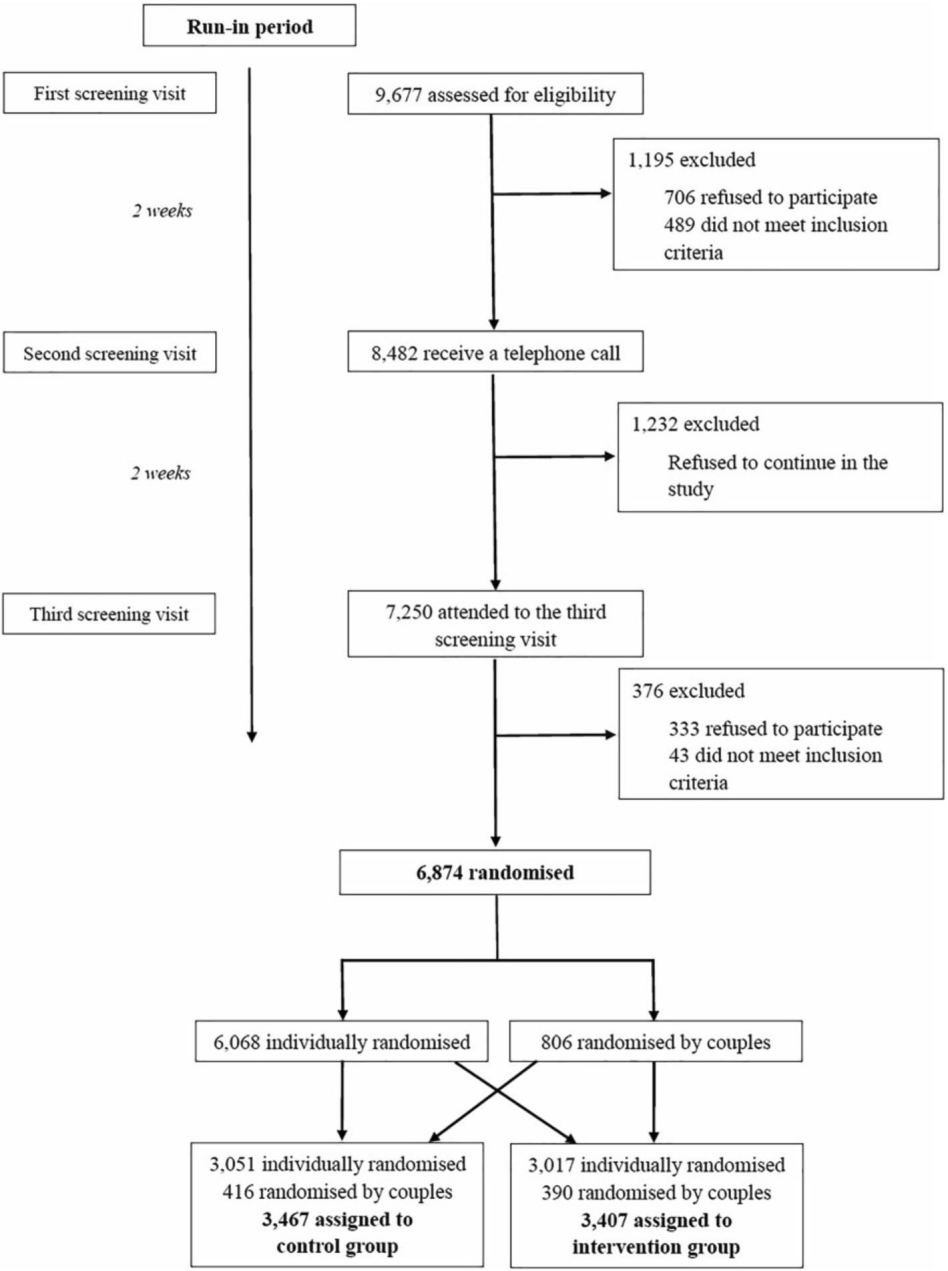
Flow chart of the PREDIMED Plus trial

From October 2013 to December 2016, 6874 participants were recruited from 23 Spanish centers (Flow chart, Figure 1). The majority of baseline characteristics (6 of 10) did not differ statistically between the intervention and control groups in this sub-sample of the ongoing PREDIMED-Plus trial (*n* = 6059), selected because of self-reported changes in PA levels. More participants in the intervention group (*n* = 2097) smoked and had lower baseline levels of total PA and MVPA, compared to the control group (*n* = 3086) (Table 1).

**Table 1:** Baseline characteristics of study participants

	All (*n* = 6059)	Control (*n* = 3086)	Intervention (*n* = 2973)	*P* ^1^
Men	3117 (51.4%)	1583 (51.3%)	1534 (51.6%)	0.814
Age, y	65.0 (4.9)	65.0 (4.9)	64.9 (4.9)	0.415
Smoker	863 (14.2%)	405 (13.1%)	458 (15.4%)	0.011
Education^2^	3075 (50.8%)	1522 (49.3%)	1553 (52.2%)	0.023
BMI, kg/m^2^	32.5 (3.45)	32.5 (3.48)	32.5 (3.43)	0.948
Waist, cm	107 (9.69)	108 (9.73)	107 (9.65)	0.511
Light PA, METs min /d	63.9 [0.00;160]	63.9 [0.00;160]	63.9 [0.00;160]	0.659
MVPA, METs min /d	158 [11;360]	160 [12;375]	150 [8;349]	0.015
Total PA, METs min /d	273 [123;488]	280 [130;499]	260 [120;480]	0.012
MDS, unit	8.49 (2.68)	8.54 (2.71)	8.45 (2.65)	0.180

*BMI* body mass index, *MET* metabolic equivalent of task, *MDS* Mediterranean diet score, *MVPA* moderate-to-vigorous physical activity, *PA* physical activity

Categorical, continuous normal, and continuous non-normal distributed variables are expressed as n (proportion), mean (standard deviation), and median (interquartile range), respectively

^1^*p* value for difference between groups from t-test or chi-square test

^2^more than primary school

MVPA increased a mean of 27.2 (5.7–48.7 95% CI) METs-min/day and 123.1 (109.7–136.6 95%CI) METs-min/day in the control and intervention group, respectively. An increase in MVPA was reported in 46.9% of the control group and 62.8% of the intervention group.

Total-PA, light-PA, and MVPA increased in both groups. The difference in PA at one-year follow-up, compared to baseline, was greater (*p* < 0.001) in the intervention group, compared to the control group (Table 2). At the same time-point, BMI and WC decreased significantly (*p* < 0.001 for both), from 32.5 kg/m^2^ to 32.2 kg/m^2^ and from 107.5 cm to 106.4 cm, respectively, in the control group and from 32.5 kg/m^2^ to 31.1 kg/m^2^ and 107.4 cm to 103.0 cm, respectively, in the intervention group.

**Table 2:** Secular trends of physical activity in intervention (*n* = 2973) and control (*n* = 3086) groups^1^

	Baseline	6 months	12 months	*p* ^2^	*p* ^3^
*Total PA (METs min/day)*					
-Intervention					
Absolute values	228 (0.48)	338 (0.48)	359 (0.48)	< 0.001	< 0.001
Relative change	Ref.	1.06 (1.03 to 1.10)	1.57 (1.51 to 1.64)		
-Control					
Absolute values	247 (0.52)	262 (0.52)	277 (0.52)	< 0.001	
Relative change	Ref.	1.06 (1.02 to 1.10)	1.12 (1.08 to 1.17)		
*Light PA (METs min/day)*					
-Intervention					
Absolute values	101 (0.49)	120 (0.49)	127 (0.49)	< 0.001	0.064
Relative change	Ref.	1.06 (1.01 to 1.10)	1.25 (1.19 to 1.31)		
-Control					
Absolute values	103 (0.50)	113 (0.50)	121 (0.50)	< 0.001	
Relative change	Ref.	1.06 (1.02 to 1.11)	1.17 (1.11 to 1.23)		
*Moderate-to-vigorous PA (METs min/day)*					
-Intervention					
Absolute values	126 (0.73)	219 (0.73)	231 (0.73)	< 0.001	< 0.001
Relative change	Ref.	1.05 (1.00 to 1.11)	1.82 (1.71 to 1.95)		
-Control					
Absolute values	142 (0.80)	154 (0.80)	160 (0.80)	0.002	
Relative change	Ref.	1.04 (0.98 to 1.10)	1.12 (1.05 to 1.20)		

^1^General estimating equation models adjusted for sex, age, education, smoking, adherence to an energy-restricted Mediterranean diet, and baseline body mass index (BMI) were used to analyze the effect of the intervention on secular trends of leisure-time physical activity in comparison with the control group. Leisure-time PA data were log-transformed for analysis. Absolute values are presented in mean and standard error (SE) and relative change in exponential beta coefficient (95% confidence interval)

^2^p for linear trend within groups

^3^p between group comparison (group*time interaction)

Table 3 shows the one-year PA trend in the intervention and control groups, stratified by sex (men/women), age (≤65y years / > 65 years), BMI (< 30.0 kg/m^2^ / ≥30 kg/m^2^), and educational level (primary education or less/more than primary education). Total PA increased in all of these strata in both groups, but significant differences between the two groups were observed in each stratum. In contrast, the increase in MVPA was significantly higher in the intervention group compared to the control group in 6 strata –men, women, both age strata, and both educational levels– but did not differ according to BMI.

**Table 3:** Secular trends of physical activity according to intervention (*n* = 2793) and control (*n* = 3086) group stratified by sex, age, educational level, and body mass index^1^

		Baseline	6 months	12 months	P^2^	P^3^
*Total PA (METs min/day)*						
Men						
- Intervention	Absolute values	263 (0.65)	397 (0.65)	409 (0.65)	< 0.001	< 0.001
	Relative change	Ref.	1.03 (0.99 to 1.08)	1.56 (1.47 to 1.65)		
- Control	Absolute values	294 (0.73)	306 (0.73)	332 (0.73)	< 0.001	
	Relative change	Ref.	1.08 (1.03 to 1.14)	1.13 (1.07 to 1.19)		
Women						
- Intervention	Absolute values	195 (0.65)	284 (0.65)	310 (0.66)	< 0.001	< 0.001
	Relative change	Ref.	1.09 (1.04 to 1.15)	1.60 (1.50 to 1.69)		
- Control	Absolute values	205 (0.70)	221 (0.71)	229 (0.71)	0.001	
	Relative change	Ref.	1.04 (0.98 to 1.10)	1.12 (1.05 to 1.19)		
*Light PA (METs min/day)*						
Men						
- Intervention	Absolute values	105 (0.68)	124 (0.68)	129 (0.68)	< 0.001	0.147
	Relative change	Ref.	1.04 (0.97 to 1.10)	1.23 (1.15 to 1.32)		
- Control	Absolute values	107 (0.70)	116 (0.71)	125 (0.71)	< 0.001	
	Relative change	Ref.	1.08 (1.02 to 1.15)	1.17 (1.09 to 1.25)		
Women						
- Intervention	Absolute values	98 (0.65)	116 (0.65)	124 (0.65)	< 0.001	0.226
	Relative change	Ref.	1.07 (1.01 to 1.14)	1.27 (1.19 to 1.36)		
- Control	Absolute values	100 (0.66)	111 (0.66)	116 (0.66)	< 0.001	
	Relative change	Ref.	1.04 (0.98 to 1.11)	1.17 (1.09 to 1.25)		
*MVPA (METs min/day)*						
Men						
- Intervention	Absolute values	150 (0.98)	264 (0.98)	273 (0.98)	< 0.001	< 0.001
	Relative change	Ref.	1.03 (0.96 to 1.11)	1.82 (1.66 to 1.98)		
- Control	Absolute values	174 (1.08)	187 (1.08)	196 (1.08)	0.014	
	Relative change	Ref.	1.05 (0.97 to 1.14)	1.13 (1.03 to 1.23)		
Women						
- Intervention	Absolute values	103 (1.04)	177 (1.04)	190 (1.04)	< 0.001	< 0.001
	Relative change	Ref.	1.07 (0.99 to 1.17)	1.84 (1.66 to 2.04)		
- Control	Absolute values	113 (1.12)	123 (1.13)	126 (1.12)	0.070	
	Relative change	Ref.	1.03 (0.94 to 1.12)	1.12 (1.01 to 1.24)		
*Total PA (METs min/day)*						
Age, ≤ 65 years						
- Intervention	Absolute values	216 (0.66)	337 (0.66)	353 (0.66)	< 0.001	< 0.001
	Relative change	Ref.	1.05 (1.00 to 1.10)	1.64 (1.54 to 1.74)		
- Control	Absolute values	241 (0.72)	261 (0.72)	284 (0.72)	< 0.001	
	Relative change	Ref.	1.09 (1.03 to 1.14)	1.18 (1.11 to 1.24)		
Age, > 65 years						
- Intervention	Absolute values	242 (0.64)	340 (0.64)	365 (0.64)	< 0.001	< 0.001
	Relative change	Ref.	1.07 (1.03 to 1.12)	1.51 (1.42 to 1.60)		
		Baseline	6 months	12 months	P^2^	P^3^
- Control	Absolute values	253 (0.72)	261 (0.72)	270 (0.72)	0.085	
	Relative change	Ref.	1.03 (0.98 to 1.09)	1.07 (1.00 to 1.13)		
*Light PA (METs min/day)*						
Age, ≤ 65 years						
- Intervention	Absolute values	95 (0.69)	115 (0.69)	122 (0.70)	< 0.001	0.102
	Relative change	Ref.	1.06 (1.00 to 1.13)	1.28 (1.20 to 1.37)		
- Control	Absolute values	100 (0.65)	112 (0.65)	116 (0.65)	< 0.001	
	Relative change	Ref.	1.03 (0.97 to 1.10)	1.16 (1.09 to 1.24)		
Age, > 65 years						
- Intervention	Absolute values	109 (0.66)	126 (0.66)	132 (0.66)	< 0.001	0.269
	Relative change	Ref.	1.05 (0.98 to 1.12)	1.21 (1.14 to 1.30)		
- Control	Absolute values	107 (0.68)	115 (0.68)	126 (0.68)	< 0.001	
	Relative change	Ref.	1.10 (1.03 to 1.16)	1.18 (1.10 to 1.26)		
*MVPA (METs min/day)*						
Age, ≤ 65 years						
- Intervention	Absolute values	123 (0.95)	222 (0.95)	229 (0.95)	< 0.001	< 0.001
	Relative change	Ref.	1.03 (0.96 to 1.11)	1.87 (1.71 to 2.04)		
- Control	Absolute values	141 (1.06)	162 (1.06)	172 (1.06)	< 0.001	
	Relative change	Ref.	1.06 (0.98 to 1.15)	1.22 (1.11 to 1.33)		
Age, > 65 years						
- Intervention	Absolute values	131 (1.06)	216 (1.06)	232 (1.06)	< 0.001	< 0.001
	Relative change	Ref.	1.07 (0.99 to 1.16)	1.78 (1.61 to 1.96)		
- Control	Absolute values	144 (1.17)	145 (1.18)	147 (1.17)	1.00	
	Relative change	Ref.	1.02 (0.93 to 1.11)	1.02 (0.93 to 1.13)		
*Total PA (METs min/day)*						
BMI < 29.9 kg/m^2^						
- Intervention	Absolute values	278 (0.79)	386 (0.79)	405 (0.79)	< 0.001	< 0.001
	Relative change	Ref.	1.05 (0.99 to 1.11)	1.46 (1.35 to 1.56)		
- Control	Absolute values	287 (0.88)	295 (0.88)	313 (0.88)	0.032	
	Relative change	Ref.	1.06 (1.00 to 1.13)	1.09 (1.02 to 1.17)		
BMI ≥ 30 kg/m^2^						
- Intervention	Absolute values	211 (0.57)	322 (0.57)	343 (0.57)	< 0.001	< 0.001
	Relative change	Ref.	1.06 (1.03 to 1.11)	1.62 (1.54 to 1.71)		
- Control	Absolute values	233 (0.63)	250 (0.63)	265 (0.63)	< 0.001	
	Relative change	Ref.	1.06 (1.01 to 1.11)	1.14 (1.08 to 1.19)		
*Light PA (METs min/day)*						
BMI < 29.9 kg/m^2^						
- Intervention	Absolute values	107 (0.87)	124 (0.88)	127 (0.87)	< 0.001	0.527
	Relative change	Ref.	1.03 (0.94 to 1.12)	1.19 (1.08 to 1.30)		
- Control	Absolute values	104 (0.94)	111 (0.94)	120 (0.94)	0.004	
	Relative change	Ref.	1.08 (1.00 to 1.17)	1.16 (1.05 to 1.27)		
BMI ≥ 30 kg/m^2^						
- Intervention	Absolute values	99 (0.56)	119 (0.56)	126 (0.56)	< 0.001	0.073
	Relative change	Ref.	1.07 (1.01 to 1.12)	1.27 (1.20 to 1.35)		
- Control	Absolute values	103 (0.59)	114 (0.59)	121 (0.59)	< 0.001	
	Relative change	Ref.	1.06 (1.01 to 1.12)	1.17 (1.11 to 1.24)		
*MVPA (METs min/day)*						
BMI < 29.9 kg/m^2^						
- Intervention	Absolute values	160 (1.18)	251 (1.18)	274 (1.18)	< 0.001	0.001
	Relative change	Ref.	1.09 (0.99 to 1.20)	1.72 (1.53 to 1.93)		
- Control	Absolute values	171 (1.41)	189 (1.41)	205 (1.41)	0.003	
	Relative change	Ref.	1.08 (0.98 to 1.20)	1.20 (1.07 to 1.34)		
BMI ≥ 30 kg/m^2^						
- Intervention	Absolute values	115 (0.89)	208 (0.89)	215 (0.89)	< 0.001	< 0.001
	Relative change	Ref.	1.04 (0.97 to 1.11)	1.87 (1.73 to 2.03)		
- Control	Absolute values	133 (0.97)	142 (0.97)	145 (0.97)	0.065	
	Relative change	Ref.	1.02 (0.95 to 1.10)	1.09 (1.01 to 1.19)		
*Total PA (METs min/day)*						
Primary education or less						
- Intervention	Absolute values	230 (0.67)	333 (0.67)	361 (0.67)	< 0.001	< 0.001
	Relative change	Ref.	1.09 (1.04 to 1.14)	1.57 (1.48 to 1.67)		
- Control	Absolute values	242 (0.74)	260 (0.74)	273 (0.74)	< 0.001	
	Relative change	Ref.	1.05 (1.00 to 1.11)	1.13 (1.06 to 1.20)		
More than primary education						
- Intervention	Absolute values	226 (0.64)	343 (0.64)	356 (0.64)	< 0.001	< 0.001
	Relative change	Ref.	1.04 (0.99 to 1.09)	1.58 (1.49 to 1.67)		
- Control	Absolute values	252 (0.71)	263 (0.71)	282 (0.71)	< 0.001	
	Relative change	Ref.	1.07 (1.02 to 1.13)	1.12 (1.06 to 1.18)		
*Light PA (METs min/day)*						
Primary education or less						
- Intervention	Absolute values	105 (0.70)	124 (0.70)	129 (0.70)	< 0.001	0.170
	Relative change	Ref.	1.04 (0.97 to 1.11)	1.23 (1.14 to 1.32)		
- Control	Absolute values	107 (0.67)	115 (0.67)	123 (0.67)	< 0.001	
	Relative change	Ref.	1.06 (1.00 to 1.13)	1.15 (1.07 to 1.23)		
More than primary education						
- Intervention	Absolute values	98 (0.64)	116 (0.64)	125 (0.64)	< 0.001	0.355
	Relative change	Ref.	1.07 (1.01 to 1.14)	1.27 (1.19 to 1.36)		
- Control	Absolute values	99 (0.71)	112 (0.71)	119 (0.71)	< 0.001	
	Relative change	Ref.	1.06 (1.00 to 1.13)	1.19 (1.11 to 1.28)		
*MVPA (METs min/day)*						
Primary education or less						
- Intervention	Absolute values	128 (1.03)	208 (1.03)	220 (1.03)	< 0.001	< 0.001
	Relative change	Ref.	1.06 (0.98 to 1.15)	1.73 (1.56 to 1.91)		
- Control	Absolute values	132 (1.17)	144 (1.17)	152 (1.17)	0.008	
	Relative change	Ref.	1.05 (0.97 to 1.15)	1.16 (1.05 to 1.27)		
More than primary education						
- Intervention	Absolute values	125 (0.99)	230 (0.99)	240 (0.99)	< 0.001	< 0.001
	Relative change	Ref.	1.04 (0.97 to 1.12)	1.92 (1.75 to 2.10)		
- Control	Absolute values	154 (1.05)	164 (1.05)	168 (1.05)	0.128	
	Relative change	Ref.	1.02 (0.94 to 1.11)	1.09 (1.00 to 1.19)		

^1^General estimating equation models adjusted for sex, age, education, smoking, adherence to an energy-restricted Mediterranean diet, and baseline BMI were used to analyze the effect of the intervention on secular trends of leisure-time physical activity in comparison with the control group. Leisure-time physical activity data were log-transformed for analysis. Absolute values are presented in mean and standard error (SE) and relative change in exponential beta coefficient (95% confidence interval)

*BMI* body mass index, *MET* metabolic equivalent of task, *MDS* Mediterranean diet score, *MVPA* moderate to vigorous physical activity, *PA* physical activity

^2^p for linear trend within groups

^3^p between-group comparison (group*time interaction)

**Fig. 2:**
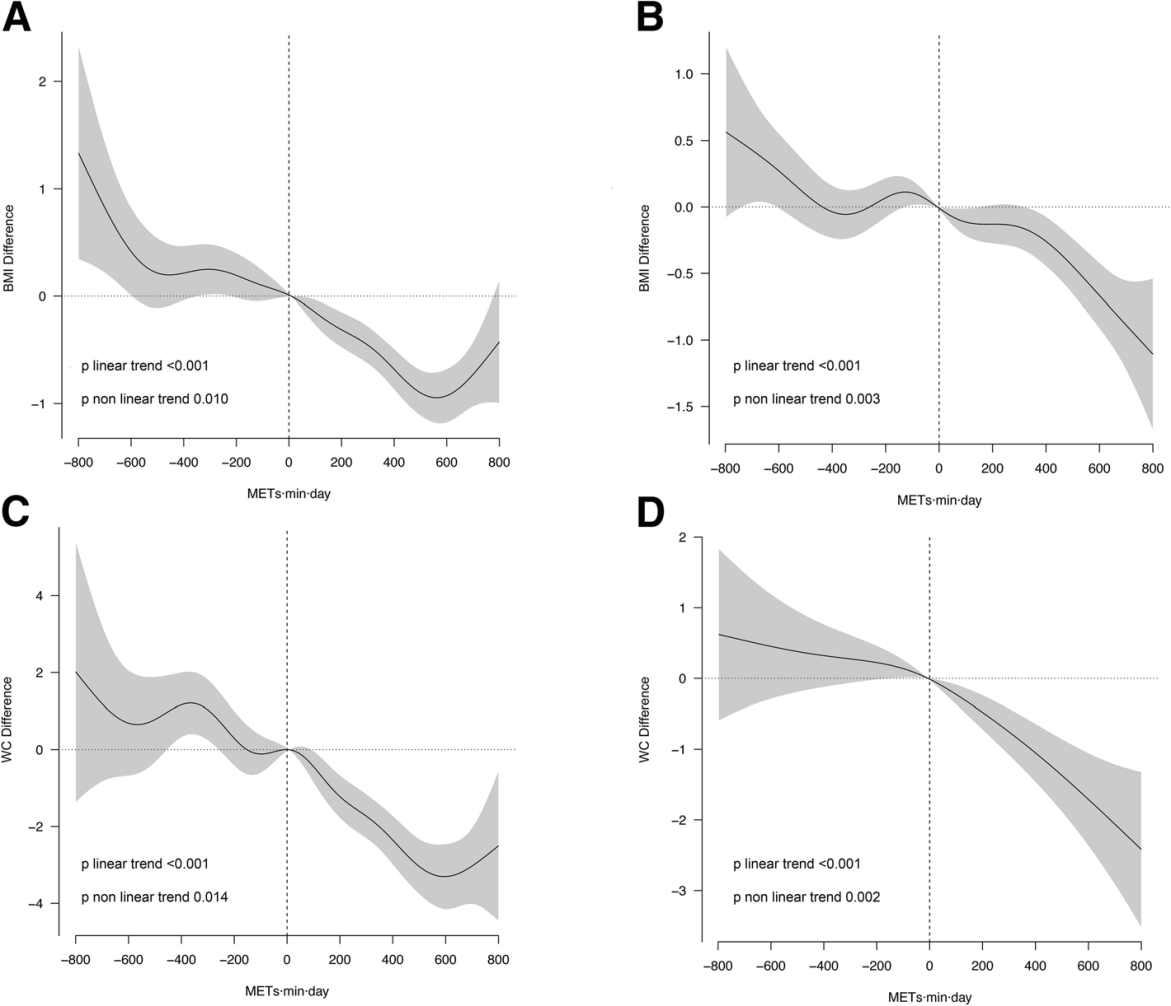
Dose-effect association between one-year differences in moderate/vigorous physical activity and one-year differences in body mass index (BMI) and waist circumferences. **a**: BMI intervention group; **b**: BMI: control group; **c**: Waist circumferences intervention group; **d**: Waist circumferences control group. All models were adjusted for sex, age, smoking, educational level, and baseline value of the corresponding anthropometric variable. Moderate-to-vigorous PA (MVPA) was measured in METs·min/day

Figure 2 shows the dose-response relationship between one-year changes in MVPA and changes in BMI and WC in each group. In both groups, BMI and WC decreased significantly (*p* < 0.001) with increasing MVPA, compared with participants who reported no changes in MVPA.

## Discussion

Participants in the personalized PA intervention program in the ongoing PREDIMED-Plus trial significantly increased all levels of PA studied after the first year of intervention; a modest but significant increase was also found in the control group. The increase of total PA and MVPA in the intervention group was significantly greater than that in the control group. Furthermore, increased MVPA was associated with a decrease in BMI and WC in both groups.

There is abundant evidence on the effectiveness of intervention programs aimed to improve PA behaviors in older adults [24]; a recently published meta-analysis found a significant difference of 73 more minutes of PA per week in the intervention versus the control group [10]. However, the heterogeneity in study design makes it difficult to determine which intervention strategies and components exert this desirable effect on PA behaviors. There is evidence that setting tailored intervention goals to specifically address questions of when, how, and where the participant is able to engage in PA are a promising approach to improvement of PA behaviors [24]. The PREDIMED-Plus PA intervention program was designed to support tailored goal-setting not only for the type of PA but also where and how the activity is feasible. It includes additional intervention components such as problem-solving, self-monitoring, feedback, informational materials, and motivation. PREDIMED-Plus trial participants are older adults with overweight or obesity and high cardiometabolic risk. Therefore, setting a daily walking goal was the first choice to increase aerobic activity in this population. As an incentive to complete individually tailored walking goals, intervention group participants received a pedometer, a motivational tool that has been shown to promote PA and consequently improve health [25–27]. The 52% increase in daily MVPA observed in the intervention group might be partially explained by the motivational effect of the pedometer.

An increase of 10% in daily MVPA was observed in the control group, although PA was not specifically promoted to this group; only MedDiet adherence was emphasized, and without restrictions on energy intake. A healthy change in other lifestyle determinants such as PA could be an additional effect of the usual clinical counseling; alternatively, trial effect (either contamination of some portion of the control group or the influence of participation in a study) could be responsible for the slight increase in MVPA.

A recent meta-analysis of pooled data on the effect of walking on cardiovascular risk factors showed a significant reduction in systolic and diastolic blood pressures and anthropometric surrogate markers of body fat [28]. In the present study, BMI and WC measurements significantly decreased as MVPA levels increased, with a comparable effect size in both groups; however, the mean increase in MVPA was considerably higher in the intervention group. Together with the concurrent dietary caloric restriction in the intervention group, this drastic increase in MVPA would explain, at least partly, the significant difference between the study groups in BMI and WC after 1 year of intervention.

Finally, we addressed the question of whether a particular subgroup of participants benefitted less from the first year of the PREDIMED-Plus PA intervention program. Evidence from the English Longitudinal Study of Ageing [29] indicates that being younger, male, a non-smoker, and of normal weight are predictors for being continuously physically active over 10 years. Furthermore, a higher educational level is associated with more PA engagement in older adults [30, 31]. In the present study, these moderators did not meaningfully influence the effectiveness of the PREDIMED-Plus PA intervention, perhaps due to the affordability of walking, the main activity that was promoted. An increase in the daily duration of walking did not imply additional costs such as joining a fitness club or the purchase of exercise-specific clothing. Therefore, adherence was feasible independently of economic status. Previous reports have shown that economic costs of adherence to a PA intervention program are an important barrier to participation, especially for individuals with low income [32].

A limitation of the present study was the use of self-reported data to evaluate the effectiveness of the PA intervention. Recall and reporting biases are inherent limitations of self-reported data. Furthermore, it has been shown that data from questionnaires overestimate the engagement in PA, compared to objective measurement by accelerometer [33]. However, it is reasonable to assume that these biases have similar effects in intervention and control groups. A further limitation was that the present analysis was not based on intention-to-treat. Tailored recommendations were focused on walking, assumed to be an affordable activity for all participants, as the first choice to increase aerobic activity. However, program affordability was not assessed. The strengths of the present study were the clinical trial design, repeated data collection, standardized measurements of anthropometric variables, and the relatively large sample size. In a multi-component intervention, we cannot rule out the possibility that the dietary component also had an effect on the reported PA outcomes, and vice versa. Future analysis will explore possible interactions between the dietary and PA components.

The study results showed the effectiveness of the PREDIMED-Plus PA intervention to increase daily PA in older adults. Implementation of this program in clinical practice would be an important step to combat the increasing prevalence of physical inactivity. Most importantly, this PA intervention program is affordable for participants.

## Conclusion

The PREDIMED-Plus PA intervention program increased PA in older adults at high risk of cardiovascular disease after 1 year of intervention. This increase was not affected by potential moderators analyzed: sex, age, education level, and obesity.

## Abbreviations

BMI: Body mass index
GEE: Generalized estimating equation
MedDiet: Mediterranean diet
MET: Metabolic Equivalent of Task
MVPA: Moderate-to-vigorous PA
PA: Physical activity
SD: Standard deviation
WC: Waist circumference
